# Breathing for answering: the time course of response planning in conversation

**DOI:** 10.3389/fpsyg.2015.00284

**Published:** 2015-03-12

**Authors:** Francisco Torreira, Sara Bögels, Stephen C. Levinson

**Affiliations:** ^1^Language and Cognition Department, Max Planck Institute for PsycholinguisticsNijmegen, Netherlands; ^2^Donders Institute for Brain, Cognition and Behaviour, Radboud UniversityNijmegen, Netherlands

**Keywords:** breathing, turn-taking, conversation, language planning, language production, speech planning, speech production, turn projection

## Abstract

We investigate the timing of pre-answer inbreaths in order to shed light on the time course of response planning and execution in conversational turn-taking. Using acoustic and inductive plethysmography recordings of seven dyadic conversations in Dutch, we show that pre-answer inbreaths in conversation typically begin briefly after the end of questions. We also show that the presence of a pre-answer inbreath usually co-occurs with substantially delayed answers, with a modal latency of 576 vs. 100 ms for answers not preceded by an inbreath. Based on previously reported minimal latencies for internal intercostal activation and the production of speech sounds, we propose that vocal responses, either in the form of a pre-utterance inbreath or of speech proper when an inbreath is not produced, are typically launched in reaction to information present in the last portion of the interlocutor's turn. We also show that short responses are usually made on residual breath, while longer responses are more often preceded by an inbreath. This relation of inbreaths to answer length suggests that by the time an inbreath is launched, typically during the last few hundred milliseconds of the question, the length of the answer is often prepared to some extent. Together, our findings are consistent with a two-stage model of response planning in conversational turn-taking: early planning of content often carried out in overlap with the incoming turn, and late launching of articulation based on the identification of turn-final cues.

## Introduction

Conversation is the core ecological niche for language—it is where language is learnt and most heavily used. Conversation is characterized by the rapid alternation of speakers, who each take mostly small turns at talk, generally avoid vocal overlap, and minimize the gap between turns (Sacks et al., 1974). This behavior appears to be, with minor wrinkles, universal in character (Stivers et al., 2009). Despite the universality and stability of this behavior, conversational turn-taking has figured little in theories about language processing, even though it poses a fundamental puzzle for them. Reported average inter-turn gap durations in the literature fall between 0 and 300 ms (e.g., De Ruiter et al., 2006; Stivers et al., 2009; Heldner and Edlund, 2010), but the latencies in language planning for production are much longer: it takes 600–1200 ms (depending on word frequency) to begin labeling a picture of an object from the moment it becomes visible (Levelt et al., 1999; Indefrey and Levelt, 2004), and it takes 1500 ms to begin producing a simple sentence describing an action picture (Griffin and Bock, 2000). Even allowing for contextual priming and facilitation, these latencies are substantial. This would seem to rule out the idea that participants simply respond to turn ends: the fastest human reaction times are of the order of 100–200 ms and the minimal latency reported for a pre-rehearsed syllable is 210 ms (Fry, 1975). Moreover, the speech signal has many brief moments of silence related to the ongoing linguistic signal (e.g., stop consonant closures), often lasting a similar duration to inter-turn gaps. So one could not recognize a silent gap as a gap before approximately 100–200 ms which, combined with minimal reaction time latency, would yield a conversational gap of 300–400 ms. These figures leave no time for the 500–1200 ms planning latencies of speech production discussed above, so the paradox of quick responses using a slow production system persists.

A plausible solution to the paradox is that, as foreseen in Sacks et al. (1974), responders often predict the content of the incoming turn, which allows them to begin planning a relevant response in advance of the turn end. The question still remains how listeners know *when* to articulate their response without causing unwanted overlap or long silent gaps (which may be semiotically loaded; cf. Kendrick and Torreira, 2015). Two possibilities can be envisaged. First, as proposed in a long tradition of observational studies (e.g., Duncan, 1972; Wells and MacFarlane, 1998; Caspers, 2003; Local and Walker, 2012), responders may launch articulation upon identifying turn-final cues (e.g., phrase-final melodic patterns, final lengthening, specific bodily gestures) occurring in the last syllables of their interlocutor's turn. This strategy could produce short gaps of 100 or 200 ms, only if at least the initial linguistic material of the responder's turn is ready to be articulated by the time the interlocutor's turn comes to an end.

An alternative option is that responders not only predict the content of incoming turns well in advance, but also estimate their timing on the basis of this prediction, and adjust the time course of their production planning based on such temporal estimation. In support of this view, for instance, De Ruiter et al. (2006) doubt that turn-final cues such as phrase-final intonation patterns are of any use for purposes of turn-taking, since they may occur too late in the turn to allow the listener to anticipate its end. Along the same lines, Magyari and de Ruiter (2012) state that it is very plausible that listeners know more than half a second in advance that a turn is going to end. Based on the results of a gating experiment, they propose that listeners make predictions in advance about which words and how many words will follow a partially heard turn, and that they use this prediction in order to estimate the remaining duration of that turn.

**Figure 1:**
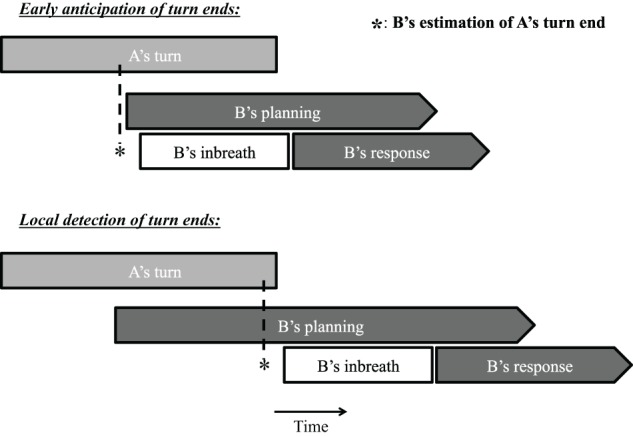
**Schematic representation of two possible response production mechanism involving a pre-utterance inbreath (see text for details)**.

In this article, we explore the time course of response planning in conversation by focusing on an ancillary source of information about language production neglected so far in psycholinguistic discussions of turn-taking, namely, breathing. Several studies have identified a relationship between breathing behavior and utterance duration, indicating that breathing can be informative about the scope of language planning (e.g., Winkworth et al., 1995; Whalen and Kinsella-Shaw, 1997; Fuchs et al., 2013; Rochet-Capellan and Fuchs, 2013). Fuchs et al. (2013) investigated several speech planning parameters, including inhalation depth and inhalation duration, using read materials varying in length and syntactic complexity, and found that inhalation depth and duration were positively correlated with utterance length. Using a corpus of spontaneous conversation, Rochet-Capellan and Fuchs (2013) also observed positive correlations between utterance length and inbreath depth and duration. Given that pre-utterance inbreaths are indicative of the length of upcoming utterances, and that in spontaneous conversation they usually take over half a second to complete (McFarland, 2001), their timing with respect to an interlocutor's turn end in a turn-taking situation may offer interesting insights into the time course of the response planning process. If listeners estimate the timing of turn ends half a second or more in advance of the turn end to time their own response, for instance by predicting the final words of a turn and their duration (cf. Magyari and de Ruiter, 2012), we should observe that they often inhale well in advance of turn ends so that their response can be produced at the right moment (much like singers and wind instrument players do in advance of their musical entries as specified in the score). If, on the other hand, responders typically determine the position of turn ends on the basis of turn-final information, we should observe that pre-utterance inbreaths tend to be taken close to the end of the interlocutor's turn, and that answers preceded by an inbreath are substantially delayed compared to answers produced on residual breath. These two alternative mechanisms, early anticipation vs. local detection of turn ends, are presented schematically in Figure 1.

A relevant issue concerning the design of this study is whether we should investigate all conversational turns in a corpus across the board, or whether we should focus instead on a specific, more controlled, conversational context. While the first approach has the advantage of potentially producing more generalizable results, it may prevent us from uncovering relevant trends in the data due to uncontrolled sources of variability. For instance, contexts in which floor changes are optional involve the complexity of deciding who will be the next speaker, which will affect the timing of the next turn in unpredictable ways. To overcome such difficulties, we have decided to focus on inbreaths taken before answers in question-answer sequences, in which a conversational response is explicitly requested by the current speaker. In this sense, question-answer sequences provide an optimal conversational context in which to begin studying the time course of language planning during conversational turn-taking (cf. Stivers et al., 2009). Moreover, question-answer sequences can be identified in a reasonably objective way on the basis of the morphosyntactic and intonational marking of questions, and of the recognizability of following turns as relevant answers (cf. Stivers and Enfield, 2010). Finally, and importantly, question-answer sequences are one of the most common action sequences in spontaneous conversation, and can therefore provide a sufficient number of observations in a medium-sized corpus such as the one used in this study (see Section Material and Data Extraction).

Because we intend to use pre-utterance inbreaths as indicators of the time course and scope of language planning, we will first assess whether breathing behavior is related to utterance length in our data, as found in previous studies. We will also need to control for the role of other communicative factors that may be at play in conversational data. It is possible that, in conversation, pre-utterance inbreaths function as meaningful elements tied to the upcoming utterance, rather than as mere preparatory phases of upcoming utterances (cf. Schegloff, 2006). Kendrick and Torreira (2015) studied the timing and construction of preferred and dispreferred responses to invitations, offers, and requests (i.e., acceptances vs. rejections) in a corpus of telephone calls in English, and found that dispreferred responses tend to be preceded by an audible inbreath more often than preferred responses. It is therefore possible that in dispreferred responses, speakers want to make their pre-utterance inbreaths salient for the listener to indicate the preference status of their responses in advance, and that, for this reason, they avoid taking them in complete overlap with the interlocutor's turn. Rochet-Capellan and Fuchs (2013), also using spontaneous conversational materials, observed that utterances containing vocalized hesitations were preceded by deeper inhalations. An anonymous reviewer notes that, because of this, it is possible that pre-utterance inbreaths are also produced by speakers as indicators of hesitations and disfluencies in their upcoming utterances, and that this may constitute another reason for answerers to avoid taking inbreaths in overlap with the interlocutor's turn. In order to better assess the relationship between breathing behavior and language planning in our statistical analyses, we will take into account the preference status of the response, and the presence of disfluencies in the response.

The following section presents a description of an audiovisual corpus of spontaneous conversation in Dutch including inductive plethysmography recordings of respiratory activity, the extraction and coding of question-answer sequences from this corpus, and the measurement scheme applied to the data. Section Results then presents several statistical analyses aimed at answering the research questions discussed above, namely, (a) whether the characteristics of pre-utterance inbreaths in spontaneous conversation are related to the scope of language planning, (b) whether responses preceded by an inbreath are delayed with respect to end of the interlocutor's turn compared to responses spoken on residual breath, and (c) what the most common timing of pre-utterance inbreaths is relative to the end of the interlocutor's turn. In section Discussion, we review and interpret our findings, and sketch a turn-taking response production mechanism accounting for both the most common trends in the data and previously reported estimates of language processing latencies.

## Materials and methods

### Material and data extraction

The corpus collection procedure and its use for research purposes were approved by the Ethics Committee Faculty of Social Sciences of the Radboud University Nijmegen. The corpus collection took place in a sound-attenuated room at the Max Planck Institute for Psycholinguistics. We recorded seven dyadic conversations between Dutch male friends, all of them university students except one participant (a research assistant). The reason for only recording males is that inductive plethysmography measurements are obtained more reliably from male participants than from female participants. Each recording had a duration of around 45 min, for an approximate total of 6 h and 15 min of dyadic conversation. Participants were briefly instructed to entertain a conversation with their dyad partner while sitting on chairs placed 1.5–2 m from each other, and oriented toward each other at an angle of 120 degrees. Each participant took part in the recordings only once.

The recording equipment consisted of a high-definition camera placed in front of the speakers, Shure SM10A head-mounted microphones, and an InductotraceTM inductive plethysmography system. Each participant wore an Inductotrace band attached around his chest at the level of the axilla, each connected to one of the two channels of the Inductotrace unit, and a head mounted-microphone coupled to an amplifier. The speech and breathing signals were recorded simultaneously at a sampling frequency of 48 kHz via an A/D converter connected to a computer. The breathing signals exhibited an upward drift starting approximately 10 min into the recording. Such a drift has not been reported in previous studies using the Inductotrace system, perhaps because their recordings were much shorter than ours. In order to correct this drift, we approximated the signals with third-order polynomials using the polyfit Matlab function, and extracted their residuals. Finally, we smoothed the signals by downsampling them by a factor of 1000.

### Coding and measures

#### Data extraction

Using Elan software (Wittenburg et al., 2006), we extracted and annotated all question and answer sequences in the data, excluding those that exhibited laughter or coughing by a participant. Wh-questions were identified on the basis of the presence of interrogative pronouns or adverbs in the utterance. Polar questions were identified on the basis of their syntactic properties (i.e., subject-verb inversion) or final intonation contour (i.e., low-rising, high-rising, or rising-falling-rising). Question and answer sequences were first identified by an assistant unaware of the purposes of the study. The first two authors then checked whether the cases identified by the assistant complied with the criteria mentioned above and only retained those that did (*n* = 171). Each dyad contributed between 15 and 30 question-answer sequences (mean = 21.6) to the dataset. Each speaker contributed between 4 and 26 answers to the dataset (mean = 12.9, *SD* = 5.9). The first author then marked the beginning and end of each question and answer. At the phonetic level, the beginning and end of answers and questions were located with reference to acoustic events in the signal attributable to either a lexical item or a particle (e.g., *uhm*, *uh*). Mouth noises, clicks and breathing noises were therefore not treated as part of the questions and answers. The beginning of the question was located with reference to syntactic structure (e.g., wh-words). The end of the answer was placed at the first pause that coincided with points of completion both at the syntactic and intonational levels. All answers therefore consisted of at least one syntactically and intonationally coherent phrase. We also coded the preference status of all answers in our data. We coded as preferred responses all responses to polar questions that matched them in polarity (e.g., *yes* answers in the case of polar affirmative questions), and answers to wh-questions that provided the requested information. Dispreferred responses included all other types of responses (e.g., negative answers to polar affirmative questions, responses to wh-questions in which the responder acknowledged not knowing the relevant answer).

We then displayed the breathing signals aligned with the audio signals in ELAN software. For each question-answer sequence, we identified inbreaths (i.e., rising trajectories of the breathing signals) only if they started after the beginning of the question and before the answer (*n* = 91; 53.2%), since inbreaths that started before the question could not have been produced in response to it. The shape of answerers' breathing signals in the considered interval showed considerable variation, and, in this respect, contrast with the breathing patterns described in studies based on highly-controlled speech. The signals could be flat with a final inbreath, but also falling or rising (i.e., indicating exhalation or inhalation), or exhibit a mixture of the preceding types (e.g., initially falling or rising, then flat, and then rising in a final inbreath). Moreover, it is probable that a number of the inbreaths that fell in the considered time interval were not primarily designed for speech. They could instead have been part of initially vital or partly vital breathing cycles that happened to occur in overlap with the question and preceding the answer. Although prototypical vital and speech breathing cycles differ very clearly under highly-controlled conditions (vital cycles are said to be more symmetrical than speech cycles, i.e., with more equal inhalation and exhalation phases; McFarland, 2001), many of the breathing cycles in our spontaneous speech data had shapes that could not be straightforwardly attributed to speech preparation or vital breathing mechanisms. Given the rapid alternation of turns of uncertain length in conversation, speakers may use different strategies to preserve sufficient lung air for speaking: For example, they may take precautions to breathe early, they may halt exhalation, or they may fall back on interruption of their production to breathe midway (cf. Bailly et al., 2013, for an illustration of different types of breathing behavior in collaborative reading). Because such strategies could not always be identified in a straightforward way, we decided not to classify the inbreaths in a qualitative way. Instead, we looked for meaningful quantitative trends in the data, while keeping in mind that different kinds of breathing behaviors were present in it.

#### Inbreath annotation

**Figure 2:**
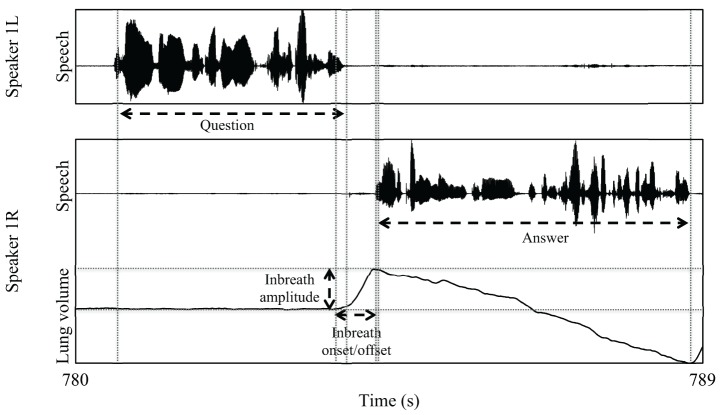
**Illustration of our measurements in a question and answer sequence exhibiting an inbreath before the answer**.

The onset and offset of each annotated inbreath was marked at the signal minimum and maximum by an assistant unaware of the purpose of the study. In some cases where there was a low plateau, the onset was located at the “elbow” located at the end of the plateau rather than at the absolute minimum. We also measured the amplitude of each inbreath, and later converted this measure to speaker-normalized *z* scores for statistical purposes (note that, since we were not particularly interested in absolute kinematic values, we did not calibrate the Inductotrace instruments). Since the amplitude values in our data are approximately normally distributed, the normalized amplitude range for each speaker should roughly extend from −2 to 2 (excluding outliers). Figure 2 illustrates our measurement scheme. From the initial timing measurements, we computed the time alignment of the beginning of the answerer's inbreath relative to the end of the question (inbreath latency from now on), and also to the start of the answer. Finally, we computed the duration of the answer, and its latency relative to the end of the question (answer latency).

### Statistical procedure

In order to investigate statistical relationships between variables of interest, we fit mixed-effects regression models using the lme4 R package (Bates et al., 2014), and perform model comparisons using the anova() function in R (R Core Team, 2014). We compare null models (including only a fixed intercept and random intercepts for each speaker), reduced models (also including any relevant covariates that may explain part of the variability of the response variable, such as the preference status of the response and the presence of disfluencies), and a full model with an added fixed predictor term for the main independent variable of interest in the analysis (e.g., answer duration in the analysis of the occurrence of pre-utterance inbreaths). In cases in which a reduced model does not improve the fit of the null model (α = 0.05), we compare the full model directly to the null model. In all models, we include interactions between the random factor speaker and any fixed predictors only if the interaction is statistically significant in a separate model comparison. Notice, however, that none of them affected the coefficients of the other factors in the model in a major way. For this reason, and for the sake of simplicity, we do not discuss them in the results section.

## Results

In this section we present several statistical analyses aimed at addressing the following research questions regarding the planning of verbal responses in spontaneous conversation (corresponding results sections below between brackets):

- Are the characteristics of pre-utterance inbreaths related to the scope of language planning? (Sections Pre-utterance Inbreaths and Answer Duration, and Inbreath Characteristics and Answer Duration).
- Are responses preceded by an inbreath delayed with respect to end of the interlocutor's turn compared to responses spoken on residual breath? (Section Pre-utterance Inbreaths and Answer Latency).
- What is the most common timing of pre-utterance inbreaths relative to the end of the interlocutor's turn? (Section Timing of Answerer's Inbreaths Relative to Question Ends).

### Pre-utterance inbreaths and answer duration

**Figure 3:**
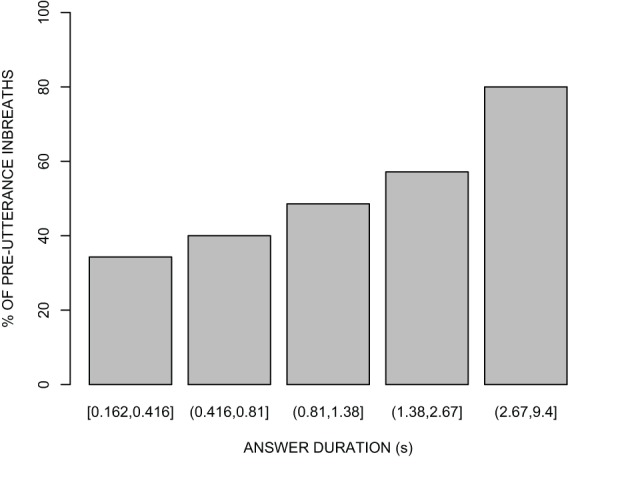
**Percentage of pre-utterance inbreaths as a function of answer duration (s) for five quantiles of approximately equal size (*n* = 35 for the lowest quantile, and *n* = 34 for all other quantiles)**.

As mentioned above, only 53.2% of the answers to questions were preceded by an inbreath. We first examined whether the presence or absence of pre-utterance inbreaths is related to the duration of the answer. Because pre-utterance inbreaths could also be affected by the preference status of the answer, and by the presence of disfluencies in the answer, we first fit two reduced logistic mixed-effects regression models with either of these two variables as fixed predictors, speaker as a random factor, and the presence of a pre-utterance inbreath as the response. The preference status of the answer did not significantly improve the fit of a null model (*p* = 0.52), and was therefore dropped from subsequent analyses. On the other hand, the presence of disfluencies in the answer provided a highly statistically significant improvement over the null model [χ^2^_(1)_ = 17.21, *p* < 0.0001], indicating that pre-utterance inbreaths are more likely before answers containing one or more hesitations. Interestingly, a model including the presence of disfluencies in the answer plus answer duration compared favorably to a model including the presence of disfluencies only [χ^2^_(1)_ = 6.38, *p* < 0.05], and indicated that pre-utterance inbreaths are more likely the longer the answer [β = 0.35, *z* = 2.31, *p* < 0.05]. This is illustrated in Figure 3, which shows the percentage of pre-utterance inbreaths as a function of answer duration. It should be noted that in the full model the β coefficient for the presence of disfluencies in the answer was not statistically significant from 0 (β = 0.76, *z* = 1.78, *p* = 0.07), perhaps due to the fact that this variable and answer duration, the other fixed predictor, were moderately correlated (*r* = 0.59). In the same way, adding the presence of disfluencies in the answer to a model with answer duration as the only fixed predictor did not result into a statistical improvement [χ^2^_(1)_ = 3.24, *p* = 0.07]. Thus, the relationship between answer duration and pre-utterance inbreaths cannot be explained away by the correlation between answer duration and the presence of disfluencies in the answer. Instead, it appears that answer duration is a better predictor of whether a pre-utterance inbreath is present than the fluency of the answer.

### Inbreath characteristics and answer duration

We then examined if, within the group of answers preceded by an inbreath, answer duration was related to inbreath depth (in speaker-normalized z-scores) and inbreath duration (in seconds; mean = 0.887 s, median = 0.72 s). We first fitted reduced models with inbreath depth and inbreath duration as responses, and either the preference status of the answer or the presence of disfluencies in the answer as fixed predictors, and observed that none of the fixed predictors was statistically related to any of the two responses (*p* > 0.05 in all comparisons with a null model). Adding answer duration to the null models did not improve its fit either for neither of the two response variables [inbreath duration: χ^2^_(1)_ = 0.16, *p* = 0.69; inbreath depth: χ^2^_(1)_ = 0.2, *p* = 0.64]. Moreover, visual inspection of the data indicated that this lack of statistical relationships was not due to outliers. Thus, contrary to previous findings (Winkworth et al., 1995; Whalen and Kinsella-Shaw, 1997; Fuchs et al., 2013), we did not observe any statistical relationship between utterance duration and the amplitude and duration of pre-utterance inbreaths.

### Pre-utterance inbreaths and answer latency

**Figure 4:**
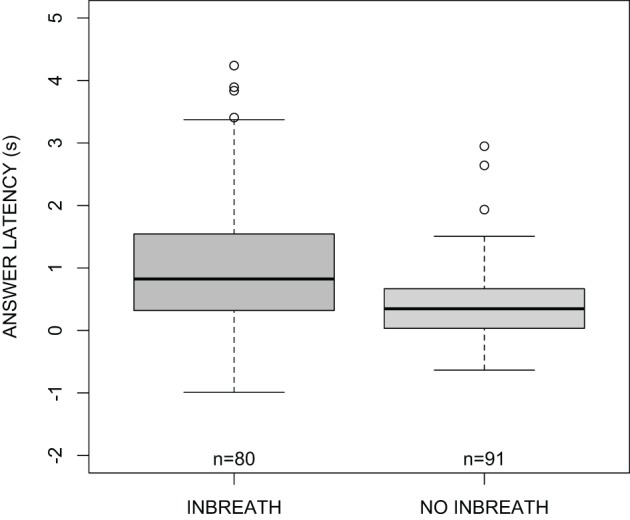
**Boxplots of answer latency (s) as a function of the presence of a pre-utterance inbreath**.

The main question that we wanted to answer in this study concerns whether answerers produce inbreaths in anticipation of question ends in order to produce answers without substantial delays (compared to answers not preceded by a pre-utterance inbreath), or if pre-utterance inbreaths occur close to turn ends, rendering responses later than those without preceding inbreaths. In order to investigate this, we first fitted reduced regression models with answer latency as the response, and either the preference status of the answer or the presence of disfluencies in the answer as a fixed predictor. None of these factors improved the null model (*p* > 0.05 in both cases). Because longer answers may take longer to plan, we also fitted a reduced model with answer duration as the fixed predictor. In this case, there was a statistical improvement over the null model [χ^2^_(1)_ = 4.61, *p* < 0.05]. Interestingly, adding the occurrence of a pre-utterance inbreath greatly improved the fit of the model [χ^2^_(1)_ = 11.2, *p* < 0.001]. As illustrated in Figure 4, answers preceded by an inbreath were substantially more delayed with respect to the end of the question than answers not preceded by an inbreath. The mean, standard deviation, median, and estimated mode for answers preceded and not preceded by an inbreath are shown in Table 1 (the mode of answer latency and other continuous variables was estimated with the function density() in R set to default parameters).

**Table 1:** **Mean and standard deviation (SD), median, and estimated modal answer latencies relative to question ends for answers preceded and not preceded by an inbreath**.

	**Answer latency**		
	**Mean (*SD*)**	**Median**	**Estimated mode**
No inbreath	459 (659) ms	347 ms	100 ms
Inbreath	998 (1008) ms	823 ms	576 ms

### Timing of answerer's inbreaths relative to question ends

**Figure 5:**
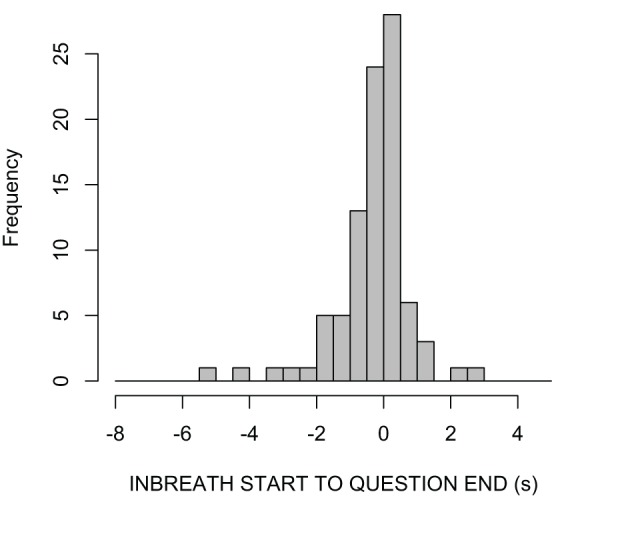
**Density plots of answerers' inbreath timings relative to question ends (s)**.

Figure 5 shows a histogram of the timing of answerer inbreaths relative to question ends. The mean and median of this measure were respectively −309 and −56 ms. Fitting the data with a continuous density function in R, the mode of the distribution was estimated at 15 ms, that is, briefly after the end of the question. The example in Figure 2, in which the answerer's inbreath is aligned close to the end of the question, is therefore representative of the most frequent cases in our data. However, there were also cases with much earlier timings, sometimes with inbreaths starting a second or more in advance of the question end. Individual inspection of such cases suggested that some of them may not have been primarily designed for speech. For instance, some of these early inbreaths were produced immediately after the end of a long turn, and were therefore likely to be conditioned more by the previous than the upcoming utterance (i.e., the answer to the question).

It is possible that inbreaths that are intended as semiotic signals, for instance announcing an upcoming dispreferred or disfluent answer, may tend to be produced in the clear rather than in overlap with the interlocutor's turn end. To investigate this, we fitted regression models with the distance from inbreath start to question end as the response, and either the preference status of the answer or the presence of disfluencies in the answer as fixed predictors. None of these two predictors provided an improvement over the null model (*p* > 0.5 in both cases). It therefore appears that the clustering of inbreath starts close to question ends is not related to the preference status or the fluency of the response.

In our data, therefore, the most typical timing of inbreaths, as captured by median and modal values, is strikingly close to the question end. This finding suggests that answerers tend to coordinate the onset of their vocal behavior, in this case an inbreath, with the end of their interlocutors' turn. However, we need to rule out an alternative interpretation, namely that the frequent alignment of inbreaths with question ends was simply caused by our annotation criteria. Recall that we annotated answerer's inbreaths only if they occurred between the beginning of the question and the beginning of the answer, that is, if they occurred either in overlap with the question or during the question-answer transition (see Figure 2 above). In a scenario in which the timing of inbreaths is random and the duration of the considered time interval is constant, we would expect a uniform distribution of inbreath timings throughout the considered time interval. However, because the considered time interval in our data was variable, it was not possible to determine the expected distribution of inbreath timings under the random timing hypothesis in a straightforward way. In order to estimate such distribution, we generated 1000 distributions of random inbreath timings within the considered time intervals in our data, and compared them with the observed distribution of inbreath timings. Because the minimum inbreath duration in our data was 210 ms, we allowed the random inbreath timings to occur randomly anywhere between the beginning of each question in the data, and 210 ms before the beginning of its answer.

**Figure 6:**
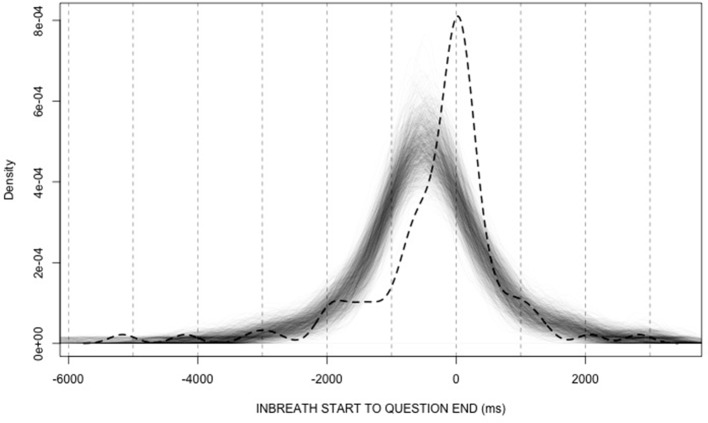
**Density plots of answerers' inbreath timings relative to question ends**. The dashed curve represents the distribution in our data (see Figure 5), while the overlaid thin lines represent randomly generated distributions.

Figure 6 shows 1000 overlaid density plots representing the randomly generated distributions (thin solid lines), along with the observed distribution (dashed line). On visual inspection, the distributions of random timings appear to have lower measures of central tendency than the observed distribution. In fact, all of the medians of the random-timing distributions were lower than the observed mode; all of the modes of the random-timing distributions were lower than the observed mode; and only 35 out of the 1000 means of random-timing distributions were equal or higher than the observed mean. Based on these proportions, the estimated probabilities that the observed median, mode, and mean were generated by a distribution of random timings are very low (i.e., median: *p* < 0.001; mode: *p* < 0.001; mean: *p* < 0.035). This suggests that the frequent alignment between answerer inbreaths and question ends observed in our data is unlikely to be random, and that it is likely to be a genuine index of coordination between questioners and answerers.

## Discussion

Our findings can be summarized as follows. First, answerers' breathing behavior in question-answer sequences in conversation is related to answer length, and this relationship cannot be explained by either the preference status of the answer or the presence of disfluencies occurring in the answer. Long answers have a greater probability of being preceded by an inbreath than short answers. In contrast, we did not find any relation between answer length and inbreath characteristics such as duration and amplitude, as reported in previous studies (Winkworth et al., 1995; Whalen and Kinsella-Shaw, 1997; Fuchs et al., 2013; Rochet-Capellan and Fuchs, 2013). Note, however, that Winkworth et al. (1995), who, like us, studied spontaneous conversations, pooled turn-internal inbreaths and inbreaths at turn transitions together, whereas we focused on question-answer sequences always involving a predictable floor transfer.

Second, answer latencies are significantly longer when an inbreath precedes the answer. The most frequent timing for answers preceded by an inbreath was around 600 ms after the question end, while the most frequent timing for answers not preceded by an inbreath was 100 ms. Third, we found that, despite significant variability in the data, there was a clear tendency for answerers to inhale briefly after the end of their interlocutors' questions, with an estimated modal offset of 15 ms. We have also shown that this tendency is not merely a chance outcome due to the durational properties of the question and answer sequences in our data. Interestingly, this finding appears to be congruous with earlier findings by McFarland (2001) and Rochet-Capellan and Fuchs (2014). McFarland (2001) studied breathing kinematics in a number of conditions, including unscripted dialogue. Using a cross-correlation method, he observed that turn exchanges were associated with a high number of significant correlations between the breathing signals of the conversation participants. These correlations were sometimes negative, indicating an anti-phase coupling, and sometimes positive, indicating an in-phase relationship. Rochet-Capellan and Fuchs (2014), also using spontaneous conversation data, did not observe a general interpersonal coordination of breathing behavior over whole conversations, but did observe specific coordinative patterns in shorter time-windows when participants exchanged turns. Although we have not examined the breathing behavior of questioners in our data, it is reasonable to assume that they often took an inbreath soon after finishing their turns. Since answerers in our data tended to inhale close to the end of questions, it is quite plausible that the breathing cycles of questioners and answerers often were in an in-phase relationship within the temporal region of the turn transition.

Our analysis of preference revealed that this factor was not a major source of variability in the breathing behavior of responders in our data. This stands in contrast with the findings of Kendrick and Torreira (2015), who found that dispreferred responses in a corpus of telephone calls tend to be preceded by an inbreath more often than preferred responses. This is perhaps due to the fact that the present study considered all pre-utterance inbreaths registered through inductive plethysmography, whereas Kendrick and Torreira could only have access to those that were audible in their acoustic data. Another possible explanation is that Kendrick and Torreira focused on a restricted number of conversational actions (responses to invitations, offers, and requests) that could take on variable linguistic forms, whereas we focused on specific linguistic forms (polar and wh-questions as defined in section Coding and Measures) that accomplished an unspecified number of actions. Finally, it is also possible that inbreaths often act as preference markers in telephone conversations, but not in face-to-face interactions. In telephone conversations, interactants do not see each other, and can only use acoustic information in order to communicate. Moreover, since speakers in telephone calls typically hold their telephones close to their lips and ears, subtle mouth noises such as inbreaths and clicks may be more efficient communicative signals in telephone calls than in face-to-face conversation.

We turn now to the interpretation of our findings. The main goal of this study was to evaluate two competing hypotheses concerning the most typical time course of language planning and production during conversational turn-taking. A model in which the articulation of one's turn relies on early prediction of turn-end timing and disregards turn-final cues (cf. De Ruiter et al., 2006) posits that listeners typically estimate the end of the incoming turn well in advance of the turn end (i.e., over 500 ms; Magyari and de Ruiter, 2012, and that they plan and launch their response in anticipation of that predicted time point. If we take into account that pre-utterance inbreaths usually last several hundred milliseconds (over 800 ms on average in our data), this model predicts that listeners will produce them in overlap with the incoming turn, so as to be able to start speaking close to the estimated turn end. On the other hand, a model consisting of early planning of content and late triggering of articulation based on turn-final cues, as discussed in Heldner and Edlund (2010), predicts that listeners will produce pre-utterance inbreaths close to the end of the interlocutor's turn, and that answers preceded by an inbreath will be delayed compared to answers produced on residual breath. Our data collected via inductive plethymosgraphy indicate that the most typical moment in which responders take a pre-utterance inbreath is briefly after the end of the question, not several hundred milliseconds in advance of its end. As a consequence of this, answers preceded by an inbreath were delayed relative to answers which were not. Our findings thus favor a model based on early prediction of content plus late triggering of articulation based on information present close to turn ends. Although we cannot discard the possibility that interlocutors use projection of turn-end timing in specific situations, our observational data suggest that late launching of vocal behavior is a more common strategy.

**Figure 7:**
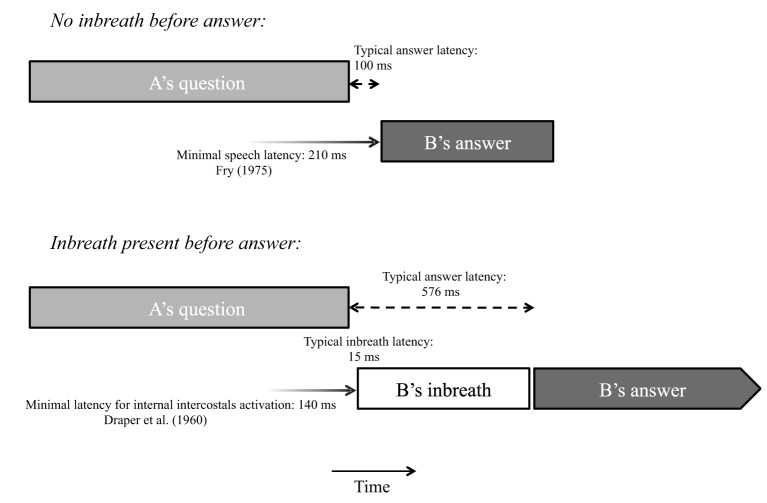
**Schematic illustration of two typical time courses of vocal behavior in question-answer sequences, along with minimal response latencies reported in previous literature**.

Since activation of the internal intercostal muscles, which are usually involved in breathing activity, requires minimally 140 ms (Draper et al., 1960), and inbreaths typically occur a few ms after the question end, we can infer that inbreath preparation for answers most often starts during the last syllable, word, or foot of the question, where phrase-final prosodic cues (e.g., final lengthening, final pitch accents, and boundary tones in a language like Dutch) and possibly other phonetic cues to turn ends (Local and Walker, 2012) become manifest. Interestingly, answers not preceded by an inbreath most frequently occurred 100 ms after the end of the question. Allowing for a minimal vocal response time of 210 ms (Fry, 1975), it can be surmised that the articulation of such answers is launched roughly at the same time as pre-utterance inbreaths when these are present. Our data therefore suggest that the launching of physical responses at turn transitions, either in the form of pre-utterance inbreaths or speech proper, typically occurs in reaction to information present in the last portion of the interlocutor's utterance. Figure 7 shows two typical time courses for vocal responses to a question in schematic form.

The fact that answerers tend to inhale more often before long answers, together with the typical alignment of inbreaths with question ends, implies that some amount of conceptual planning involving the size of the answer must already take place in overlap with the incoming question. This finding is consistent with recent EEG evidence that response preparation starts well in overlap with the incoming turn (Bögels et al., 2014). The claim that production planning significantly overlaps with comprehension processes is nevertheless puzzling, because it implies dual tasking using much of the same neural circuitry (e.g., Indefrey and Levelt, 2004; Menenti et al., 2011; Hagoort and Indefrey, 2014) and an intensive sharing of attentional resources (cf. Jongman et al., 2015). One can only speculate about how this may be possible, for example, by a rapid switching of resources between the two processes, with a gradual increase of allotted time-share to production.

The considerations on the time course of language production in conversational turn-taking presented above are based on the most typical values observed in our data, and on minimal response latencies reported in previous research. Importantly, however, we also observed a significant amount of variability in breathing and answer latencies, with relatively long overlaps and gaps accounting for a substantial portion of the data. Under the two-stage production mechanism outlined above (i.e., early planning of content overlapping with the interlocutor's turn, plus late launching of articulation based on incoming turn-final cues), such non-smooth turn transitions require further explanation. Such cases could arise when either early language planning or the launching of articulation based on turn-final cues are not carried out optimally. For instance, one common cause of speech overlap routinely mentioned in the Conversational Analysis literature (e.g., Jefferson, 1986) is that turns may contain several potential ends (i.e., transition relevance points, or TRPs) within them (e.g., “Are you coming later? To the party?”), and that listeners may time their turn with respect to one of the non-final possible turn ends (e.g., the word “later” in the previous example). Launching articulation without waiting to hear a silence at the end of the interlocutor's turn is, in fact, what our data suggest, and what our model predicts.

In cases of long inbreath latencies, the responder may not have been able to plan the initial stages of her turn (e.g., conceptual planning) early enough to determine whether she needs to take an inbreath before her turn, and launch it in response to the interlocutor's turn-final cues. This may be due to a low attentional level on the part of the speaker, or to the interlocutor's turn being unclear until its very end. In cases in which the speaker is able to complete the initial stages of language production in time to provide a smooth response, but not the later stages (e.g., phonological encoding of the beginning of her turn), she could still take an early inbreath upon identification of the turn-final cues in the interlocutor's turn, and then use her inbreath, which may stretch for several hundred milliseconds, as a buffer through which to complete the planning of the utterance.

We hope to have shown that the study of breathing can shed new and interesting light on the underlying mechanisms involved in turn-taking. The current study is limited to question-answer contexts in which answers are always produced in response to a question. We think that our conclusions regarding the answerer's breathing behavior can be expected to be valid in turn-taking contexts involving readiness to respond on the part of one of the interlocutors. However, further research should explore other conversational contexts in which floor changes may be optional (i.e., end of conversational sequences), subject to increased competition for the floor (e.g., multi-party conversation) or involving highly predictable first turns (cf. Magyari and de Ruiter, 2012), since different production mechanisms might be used in different situations. It would also be interesting to relate the breathing signal to other early signals of speech preparation obtained by direct measurement of the vocal organs via ultrasound (Drake et al., 2014; Palo et al., 2014; Schaeffler et al., 2014) or other instrumental techniques such as electromagnetic articulography. We believe this is a rich field that should be further explored.

### Conflict of interest statement

The authors declare that the research was conducted in the absence of any commercial or financial relationships that could be construed as a potential conflict of interest.
